# Differential hemodynamic characteristics of high-resistance vs. high-flow type of pulmonary artery hypertension revealed by phase-contrast MRI

**DOI:** 10.1186/1532-429X-17-S1-O69

**Published:** 2015-02-03

**Authors:** Ming-Ting Wu, Chen-Hua Wang, Chin-Chang Cheng, Wei-Chun Huang, Ming-Jyh Chern

**Affiliations:** 1 Radiology, Kaohsiung Veterans General Hospital, Kaohsiung, Taiwan; 2 Faculty of Medicine, School of Medicine, National Yang Ming University, Taipei, Taiwan; 3 Mechanical Engineering, National Taiwan University of Science and Technology, Taipei, Taiwan; 4 Cardiovascular Medicine, Kaohsiung Veterans General Hospital, Kaohsiung, Taiwan

## Background

Phase-contrast MR (PC-MR) is able to show the hemodynamic features of pulmonary artery hypertension (PAH) in comparison to normal subjects. We aimed to further characterize the subtypes of high-flow vs. high-resistance PAH using 3D PC-MR.

## Methods

Total 68 subjects were enrolled, including: Group 1, 19 normal control (38.5±16 years old, M/F=2/17); Group 2, 15 high-flow type PAH, related to untreated simple congenital heart disease (39.7±17.9 years old, M/F=4/11) and Group 3, 34 high-resistance type PAH (47.9±18.5 years old, M/F=6/28), including idiopathic PAH (N=15) and SLE-related PAH (N=19).

MRI was performed on 1.5T scanner. 2D and 3D PC-MR on the cross-section and 4D PC-MR on longitudinal axis of main pulmonary artery were performed. Data analysis was performed by Flow Quantification Analysis (MedVoxel, Vancouver, CA) for area and distensibility, flow rate and velocity, accelerative time and pulse wave velocity (PWV). An institute- developed program via MatLab^TM^ and tecplot^TM^ was used for visualization and quantification of the number and duration of vortex, and the pressure gradient and pulmonary vascular resistance (PVR).

Echocardiography estimated pulmonary artery pressure gradient (echoPASP) were performed for all subjects. Right heart catheterization for mean pulmonary artery pressure (meanPAP) was performed for Group 2 and 3 within 3 months of PC-MR.

## Results

As compared to Group 1, MPA of Group 2 and 3 showed larger cross-sectional area (P<0.001), decreased distensibility (P=0.001), slower flow velocity (P<0.001), faster PWV (p=0.005), with more (P<0.001) and longer duration (P=0.030) of vortex and higher PVR (P<0.001).(Table)

Compared to Group 2, Group 3 had lower flow rate (P=0.002), shorter accelerative time (P=0.003) and higher PVR (P=0.018).(Table)

With multiple linear regression model, we found that vortex (number and duration) and PWV of MPA predicted well of meanPAP in Group 3 (R=0.821), while only moderately in Group 2 (R=0.625). With adding the distensibility of MPA, the prediction of mean PAP improved significantly (R=0.625 to 0.978) in Group 2, while only marginally in Group 3 (R=0.821 to 0.873).

## Conclusions

PC-MR revealed overt different hemodynamics of PAH as compared to the normal subjects. In addition, several hemodynamic characteristics were distinct between the high-resistance type and high-flow type PAH. The increase of number and duration of vortical flow reflected the increase of pressure in high-resistance PAH, while the MPA distensibility reflected the hyper-dynamic flow in high-flow PAH. The discrepancy disclosed the different underlined pathophysiology between the subtypes of PAH.

## Funding

Grant: NSC 100-2314-B-010 -045 -MY3, Taiwan.

**Table 1:** Hemodynamic characterisitcs of main pulmonary artery in the three groups.

	Group 1	Group 2	Group 3
	
	Normal	High-flow PAH	High-resistance PAH
	
	N=19	N=15	N=34
Main pulmonary artery	Mean ± SD	Mean ± SD	Mean ± SD

echoPASP, mmHg	30.3 ± 15.6	74.0 ± 35.8	70.5 ± 29.2

mean PAP, mmHg		48.1 ± 27.8	41.7 ± 14.5

area, max, cm2	6.6 ± 1.7	14.4 ± 5.6	12.3 ± 5.0

distensibility, %	24.9 ± 8.7	17.9 ± 11.8	14.4 ± 9.2

flow rate, mL/min	5791.2 ± 1286.0	7143.9 ± 5393.7	4586.0 ± 1580.6

peak velocity, cm/s	46.7 ± 7.6	29.5 ± 17.5	23.3 ± 11.4

accelerate time, ms	232.0 ± 25.3	211.3 ± 44.5	171.2 ± 39.9

Pulse wave velocity, m/s	2.0 ± 1.0	3.5 ± 1.9	3.4 ± 1.5

vortex, number	3.5 ± 3.0	12.1 ± 8.2	13.0 ± 8.2

vortex, duration, s	0.17 ± 0.13	0.28 ± 0.13	0.22 ± 0.09

resistance (PVR), dyne.s.cm-5	16.3 ± 6.6	24.7 ± 14.9	39.9 ± 21.0

